# The effects of school psychological capital and achievement emotions on behavioral engagement of Chinese secondary mathematics learners: a structural equation modeling approach

**DOI:** 10.3389/fpsyg.2025.1646461

**Published:** 2025-09-29

**Authors:** Xia Kang, Shuqing Zhang, Yajun Wu

**Affiliations:** ^1^School of Mathematics and Information Science, Guangzhou University, Guangzhou, China; ^2^School of Humanities, Foshan University, Foshan, China

**Keywords:** school psychological capital, academic enjoyment, academic anxiety, behavioral engagement, secondary school students, structural equation modeling

## Abstract

**Background:**

School psychological capital (PsyCap), the positive psychological state or psychological resources displayed within school contexts, has been shown to correlate positively with achievement emotions, engagement, and subjective well-being. Few studies, however, have investigated whether achievement emotions mediate the association between school PsyCap and behavioral engagement.

**Methods:**

This study aimed to investigate the emotional underpinnings linking students’ school PsyCap to their behavioral engagement in mathematics classrooms. Self-report data on mathematics-related PsyCap resources, enjoyment, anxiety, and behavioral engagement were collected from 1135 Chinese secondary school students aged 12–15 years (M_*age*_ = 13.15, SD = 0.75). Having controlled for prior achievement and gender, structural equation modeling (SEM) was used to explore the potential mediating effect of academic enjoyment and anxiety in the relationship between school PsyCap on behavioral engagement.

**Results:**

The findings revealed that higher levels of school PsyCap and positive emotional experiences were positively correlated with greater behavioral engagement in mathematics learning. Conversely, negative emotions were negatively associated with engagement. SEM analyses indicated that achievement emotions (enjoyment and anxiety) partially mediated the association between school PsyCap and behavioral engagement.

**Conclusion:**

Students with higher levels of school PsyCap exhibited greater positive achievement emotions and lower levels of negative emotion in mathematics classes, which, in turn, were associated with higher levels of behavioral engagement. These findings highlight the importance of fostering students’ school PsyCap to enhance their emotional well-being and engagement in mathematics learning.

## 1 Introduction

The present study focused on how school PsyCap, defined as students’ positive psychological state and resources, influences behavioral engagement through academic enjoyment and anxiety. Prior research has demonstrated that school PsyCap positively impacts achievement emotions, engagement, and subjective well-being (Carmona-Halty et al., 2019; Kang et al., 2021; King et al., 2020; Li et al., 2014; Siu et al., 2014). Research has also shown the school PsyCap was positively correlated with academic performance (Datu et al., 2018; Luthans et al., 2012). However, limited attention has been directed toward understanding the mediation mechanism between school PsyCap and behavioral engagement, particularly within the context of mathematics education. Therefore, this study endeavored to investigate the association between school PsyCap and behavioral engagement in the context of mathematics classrooms, with a focus on elucidating the emotional mechanisms that underpin this relationship.

### 1.1 School psychological capital

School psychological capital refers to an individual’s positive psychological state or psychological resources that arise from endorsing four psychological capacities: hope, optimism, resilience, and self-efficacy (Luthans et al., 2004). Since PsyCap was first introduced from the industrial-organizational context to the educational domain, this construct has been hotly discussed (Carmona-Halty et al., 2019; Kang et al., 2025; Vîrgă et al., 2022). However, the measurement of school PsyCap was controversial, given that this construct was established based on the synchronicity of the academic community (Kang et al., 2021). For example, the unidimensional model regarded school PsyCap as an omnibus construct, and the value of this construct was calculated by summing up and averaging the value of each item (Li et al., 2014). In comparison, the four-factor model treats the four individual components as observational variables (Carmona-Halty et al., 2021; Datu and Valdez, 2016). Recently, the hierarchical model (or second-order model) that views both school PsyCap and its four components as latent variables has begun to gain support from scholars (Datu et al., 2018; Kang et al., 2021; King et al., 2020; King and Caleon, 2021).

Hope, self-efficacy, resilience, and optimism or the “HERO” within (e.g., see Luthans, 2012) were the four psychological resources that constitute PsyCap, each of which has been extensively discussed in educational literature. Hope pertains to the goal-related thinking orientation characterized by three components: goals, pathway, and agency (Snyder, 2000). Existing studies have shown that hope positively predicted key learning-related outcomes, including academic performance (Buckelew et al., 2008; Day et al., 2010; Marques et al., 2011; Snyder et al., 2002), emotional well-being (Ciarrochi et al., 2007), behavioral engagement (Chen et al., 2020; van Ryzin, 2011), and psychological adjustment (Du and King, 2013). Self-efficacy (or academic confidence) refers to an individual’s judgment of his or her ability to complete challenging tasks (Urdan and Schoenfelder, 2006). A considerable number of studies showed that self-efficacy is positively correlated with different indices of optimal functioning such as motivation and learning (Linnenbrink and Pintrich, 2003; Van Dinther et al., 2011), academic performance, and learning-related emotions (Putwain et al., 2013), course satisfaction (Doménech-Betoret et al., 2017), mental well-being (Andretta and McKay, 2020), and engagement (Lin, 2021; Simón et al., 2024). Besides, self-efficacy has also been found to positively correlate with life satisfaction and moderate the relationship between stress and life satisfaction (Moksnes et al., 2019). Resilience is an individual’s ability to sustain and bounce back when facing problems and adversities (Martin and Marsh, 2006). Those high in resilience would see adversities as challenges rather than threats (Symes et al., 2015). The positive effect of resilience on crucial indices of school functioning has also been extensively verified (Allan et al., 2014; Ayala and Manzano, 2018; Kotzé and Kleynhans, 2013). For example, García-Izquierdo et al. (2018) documented that resilience positively correlated with psychological health and moderated between burnout and psychological health in a sample of sophomore nursing students. Moreover, the predictive effects of resilience on academic performance (Ayyash-Abdo et al., 2016; Cai and Meng, 2025), academic satisfaction (Meneghel et al., 2019), emotional engagement (Kim et al., 2021), and psychological well-being (Voon et al., 2021) were also confirmed. Optimism pertains to an individual’s expectation of positive outcomes in their endeavors. Researchers have found that optimism positively affects academic achievement and psychological well-being. For example, in a study among high school students, Hoy et al. (2006) documented that academic optimism was positively correlated with student achievement. Taking undergraduate students as participants, Vizoso et al. (2019) reported that optimism positively affected academic performance by mitigating academic burnout. In another study with undergraduate women, Augusto-Landa et al. (2011) observed that optimism was positively associated with all dimensions of psychological well-being. Similarly, the predictive effects of optimism on engagement (Geers et al., 2009) and psychological well-being (Krok, 2015) were also confirmed.

The predictive effect of school PsyCap has also been recognized within educational contexts. For example, Siu et al. (2014) documented that PsyCap predicts school engagement directly or indirectly via intrinsic motivation among university students in Hong Kong. Similarly, in a study conducted among Filipino high school students, school PsyCap positively correlates with academic motivation, engagement, achievement, and well-being (Datu et al., 2018; Datu and Valdez, 2016). In line with these findings, research involving Chilean high school students supported the positive association between PsyCap and academic performance (Carmona-Halty et al., 2019, 2021; Carmona–Halty et al., 2019).

Based on the above review, it is evident that both school PsyCap and its four components demonstrate a positive correlation with academic engagement. However, relatively few studies have been conducted to explore the mechanism between school PsyCap and academic engagement, especially in a Mainland Chinese context. An exception was the work of King et al. (2020), who demonstrated that positive emotions partially mediate the relationship between school PsyCap and academic engagement in a sample of 71 Hong Kong secondary school students. One key limitation of this study was that it ignored the domain-specificity of emotions and academic engagement in the school context (Goetz et al., 2007; Green et al., 2007). In addition, both positive and negative achievement emotions would be experienced in the subject-related learning process (e.g., Lichtenfeld et al., 2012; Pekrun, 2006; Raccanello et al., 2013), however, only positive emotions have been shown to mediate the relationship between school PsyCap and academic engagement in their study (King et al., 2020). The present study aimed to extend this research using mathematics-related data to investigate the emotional connections (both positive and negative achievement emotions) between school PsyCap and behavioral engagement in the context of mathematics classrooms.

### 1.2 Achievement emotions

Achievement emotions, which refer to discipline-related emotions that are closely related to achievement activities or achievement outcomes (Pekrun, 2006), have drawn a growing number of educational researchers’ attention as their ubiquity and their considerable influence on learning and achievement (Pekrun and Stephens, 2010; Putwain et al., 2022; Villavicencio and Bernardo, 2016). For example, King and Gaerlan (2014) investigated the consequence of achievement emotions. They found that achievement emotions had a predictive effect on engagement and, thus, academic achievement in a sample of Filipino university students. Furthermore, the beneficial effects of positive emotions on self-efficacy, self-regulation, achievement (Villavicencio and Bernardo, 2016), intrinsic motivation and engagement (Oriol et al., 2016), coping strategies (Vierhaus et al., 2016), and learning satisfaction (Lee et al., 2021) were also confirmed.

Through a series of qualitative and quantitative studies, Pekrun et al. (2002) identified nine emotions that students most commonly experienced (i.e., enjoyment, pride, hope, relief, anxiety, boredom, hopelessness, anger, and shame) in their learning experiences. Valence, activation, and object focus are the three dimensions for assessing achievement emotions (Pekrun, 2006; Pekrun et al., 2002). According to this classification criteria, every achievement emotion can be concluded by three dimensions: valence–positive versus negative, activation–activated versus deactivated, and object focus–activity-related versus outcome-related. Concerning these three dimensions, enjoyment, for example, can be described as a positive activity-related emotion that facilitates activation.

According to the control-value theory (CVT) of achievement emotions (Pekrun, 2006), students’ achievement-related controllability and value that are experienced at school are the two most essential antecedents of their achievement emotions (Pekrun, 2006; Pekrun and Stephens, 2010). The learning environment, including environmental, situational, and individual information, plays a vital role in the arousal of achievement emotions (Forsblom et al., 2021). Previous studies concentrated mainly on the influence of environmental and situational factors on achievement emotions (e.g., Clem et al., 2021; Forsblom et al., 2021; Westphal et al., 2018), however, the impact of individual factors on achievement emotions was relatively unexplored (for exceptions, see Kang and Wu, 2021).

### 1.3 Behavioral engagement

As one subtype of academic engagement (including behavioral, affective, and cognitive) (e.g., Archambault et al., 2009; Ben-Eliyahu et al., 2018), behavioral engagement refers to students’ intensity of involvement within both academic or non-academic activities in the school context (Ben-Eliyahu et al., 2018; Chen et al., 2020). These three subtypes differ in connotation but are concurrently interrelated (Fredricks et al., 2004). Among them, behavioral engagement has been the most tremendously studied in connection with achievement-related outcomes, partly because students’ behavioral engagement can be observed in learning-related situations regarding participation and interest in academic tasks, following teacher instructions, and persistence and inhibition toward academic tasks (Chen et al., 2020; Darensbourg and Blake, 2013; Gregory et al., 2014). In addition, behavioral engagement demonstrated a more substantial predictive effect on school achievement than the other two subtypes of academic engagement. In a sample of high school students in Quebec, Archambault et al. (2009) found that only behavioral engagement was significantly correlated with dropout, while affective and cognitive engagement was not. During the primary grades, Ladd and Dinella (2009) found that the predictive contribution of behavioral engagement to long-term achievement was more potent than emotional engagement. Similarly, Wang et al. (2012) compared the contribution of these three types of engagement to academic performance and educational aspiration in a sample of African-American and European-American students in grades seven through eleven. They found that emotional engagement can only contribute to academic performance via behavioral or cognitive engagement. Furthermore, the present study was concerned with the participation dimension of academic engagement in the context of the mathematics classroom, so the key constructs referring to classroom activities–namely, behavioral engagement were chosen (e.g., Hospel et al., 2016).

Behavioral engagement was believed to affect a diverse set of desired learning-related outcomes positively. For instance, Wang and Pomerantz (2009) documented that students’ continuous behavioral engagement contributed to their school performance in a sample of American and Chinese secondary school students. In the same vein, the robust predictions of behavioral engagement on school graduation (Kieffer et al., 2013), interpersonal relationships (i.e., peer relation and teacher-student relations) (Engels et al., 2016), and student retention (Sengupta and Williams, 2021) were also confirmed. Given the self-determination theory, students’ school engagement was seen as manifesting their inner motivational resources (Reeve and Halusic, 2009; Skinner et al., 2009; Skinner and Pitzer, 2012). It plays a positive role in predicting various achievement-related outcomes.

### 1.4 The present study

**FIGURE 1:**
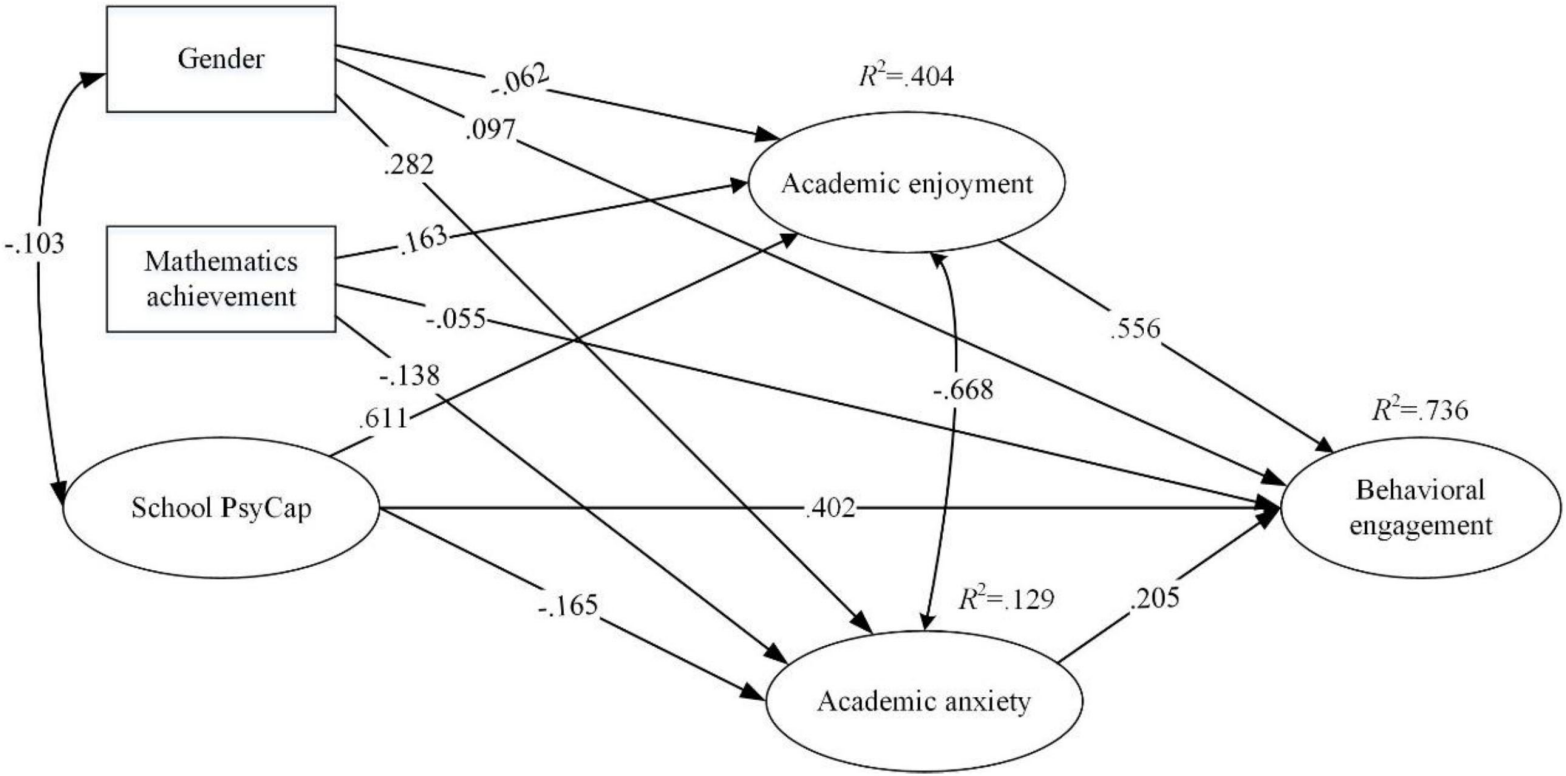
The relation between school PsyCap, academic enjoyment, academic anxiety and behavioral engagement. All the correlations and path coefficients shown in the figure are standardized and statistically significant (*p* < 0.05).

To explore the emotional connection between school PsyCap and behavioral engagement, the present study examined how school PsyCap influences enjoyment and anxiety and how these two achievement emotions, in turn, affect behavioral engagement in a sample of Chinese secondary school students in the mathematics classroom context (see Figure 1 for the mediational model). Building on the literature review, the present study posited that variability in students’ school PsyCap would lead to differential experiences of achievement emotions in the learning environment. For example, in a sample of Chinese secondary school students, Kang and Wu (2021) found that students with higher levels of school PsyCap tend to experience more positive achievement emotions (i.e., enjoyment, hope, and pride) and fewer negative emotions (i.e., anxiety, anger, shame, boredom, and hopelessness) in mathematics learning. Furthermore, the significant association between achievement emotions and academic engagement has also been demonstrated in a large body of literature (e.g., Carmona-Halty et al., 2021; King et al., 2020; King and Gaerlan, 2014; Oriol et al., 2016). Collectively, the present study posited that achievement emotions (i.e., enjoyment and anxiety) mediate the association between school PsyCap and behavioral engagement. The present study contributes to literature mainly by testing the following three hypotheses:

H1. Students’ school PsyCap will be significantly correlated with their positive and negative achievement emotions in mathematics learning.

H2. Students’ school PsyCap would be positively correlated with their behavioral engagement within the mathematics classroom.

H3. Achievement emotions–both negative and positive–serve as mediators in the relationship between school PsyCap and behavioral engagement.

## 2 Materials and methods

### 2.1 Participants and procedures

One thousand one hundred thirty-five participants from two secondary schools took part in the present study, comprising seventh graders (*N* = 557) and eighth graders (*N* = 578). The two secondary schools are in two towns in Xishuangbanna Dai Autonomous Prefecture, the extreme southwestern border of Yunnan Province, China. The mean age of participants was 13.15 years (SD = 0.75), with 574 (50.6%) males and 561 (49.4%) females. When students were invited to participate in this survey, permission was sought from students, administrators, teachers, and parents. After these consents were obtained, participants completed the questionnaire under the guidance of their headteacher. Throughout the data collection process, students were informed that their participation was voluntary, and refusal to participate would not affect their academic standing or relationships within the school.

### 2.2 Measures

#### 2.2.1 School psychological capital scale

The 15-item Chinese version of the school PsyCap scale adapted from King and Caleon’s (2021) English version was initially applied to measure the PsyCap of secondary school students in Singapore. The validity of the Chinese version of the school PsyCap scale has been verified among Chinese university students (Kang et al., 2021) and Chinese secondary school students (Kang and Wu, 2021). Consistent with existing literature (Datu et al., 2018; Kang et al., 2021; Kang and Wu, 2021; King et al., 2020; King and Caleon, 2021), a 15-item school PsyCap scale was adopted to measure the level of school PsyCap of students. Besides, the school PsyCap was viewed as a second-order construct underpinned by its four first-order components (i.e., hope, self-efficacy, resilience, and optimism). Precisely, hope was measured by four items (e.g., “I expect the best during uncertain times at school”) with a reliability of 0.68. Self-efficacy was measured by four items (e.g., “I feel confident that I can learn what is taught in school”) with a reliability of 0.68. The reliability of resilience (Cronbach’s α = 0.80) and optimism (Cronbach’s α = 0.61) was also acceptable. Four items measured resilience, and optimism was measured by three items, with example items being “I don’t let study stress get on top of me” (resilience) and “I am optimistic about my future in school” (optimism). The school PsyCap was rated on a five-point Likert scale ranging from 1 (strongly disagree) to 5 (strongly agree). Internal consistency of school PsyCap was estimated as Cronbach’s α = 0.97, indicating that the school PsyCap scale demonstrated good reliability.

#### 2.2.2 Mathematics class-related achievement emotions scale

Participants were asked to rate items adapted from Pekrun et al.’s (2011) achievement emotions questionnaire-mathematics (AEQ-M) on whether the statements fit their mathematics class-related achievement emotions. First, students’ enjoyment of their mathematics class was measured by asking them how they felt about attending mathematics classes. Participants responded to the four-item mathematics class-related enjoyment scale (e.g., “I look forward to my mathematics class” and “I enjoy my mathematics class”) on a five-point scale (1 = strongly disagree, 5 = strongly agree). A higher score indicated a more fantastic endorsement of the enjoyment construct. The reliability and validity of the enjoyment subscale in the mathematics context have been verified in existing literature, including secondary school students in China (e.g., Chen and Brown, 2018; Kang and Wu, 2021), and the internal consistency for the present study was good with Cronbach’s α = 0.83.

Second, participants’ mathematics class-related anxiety was measured by four items adapted from AEQ-M (Pekrun et al., 2011). Exemplar items include “I feel anxious in mathematics class” and “I worry that I cannot follow what is being taught in math class.” Participants rated their level of agreement on the four items of anxiety scale using a five-point scale from 1 (strongly disagree) to 5 (strongly agree). A high score indicates higher levels of anxiety. The reliability and validity of the mathematics class-related anxiety subscale have also been established in prior studies, including secondary school students in China (e.g., Kang and Wu, 2021; Lin et al., 2020), and the internal consistency of the anxiety subscale was also acceptable for the present study (Cronbach’s α = 0.66).

#### 2.2.3 Behavioral engagement scale

The behavioral engagement was assessed using four items adapted and adjusted from the Engagement vs. Dissatisfaction with Learning Questionnaire developed by Skinner et al. (2009). All items were modified and adapted to be specific to mathematics lessons or activities (e.g., “In mathematics class, I work as hard as I can”). The four items were answered on a five-point scale (1 = strongly disagree, 5 = strongly agree) such that a high value represents higher levels of behavioral participation in mathematics classes. The reliability and validity of this construct have been proved in existing literature (e.g., King and Ganotice, 2015; Putwain et al., 2018; Skinner and Chi, 2012), and the internal consistency of behavioral engagement construct in the present study was good (Cronbach’s α = 0.78).

#### 2.2.4 Covariates

Gender and prior mathematics achievement were included as covariates in the analysis. First, previous studies suggested that students’ academic engagement demonstrates gender bias, which would cause gender gaps in their levels of mathematics-related learning activities (Kessels et al., 2014; Loh, 2019; Moss-Racusin et al., 2018). For example, Nosek and Smyth (2011) reported that the mathematics-gender stereotype contributed to sex gaps in engagement and achievement in mathematics, predicting that female students would be more harmful, engage less, and gain worse grades in mathematics. The reciprocal relationship between mathematics-related behavioral engagement and academic achievement (Wang et al., 2019) indicated that the analysis should control prior achievement. Prior mathematics achievement was represented by students’ mathematics scores in the latest final examination. This exam was developed by the Education Committee of the Xishuangbanna Dai Autonomous Prefecture and administered as the final exam for all secondary school students in Xishuangbanna Dai Autonomous Prefecture in the first semester of the 2020–2021 academic year. Since gender and prior mathematics achievement have been proven to be associated with students’ mathematics-related behavioral engagement, these variables were controlled as covariates.

#### 2.2.5 Data analysis

The data analysis in the present study comprises five sequential steps. First, the complete information maximum likelihood (FIML) method, conducted in Mplus 8.3 (Muthén and Muthén, 2017), was used to handle the missing data given that FIML would produce trustworthy and unbiased estimates (Nicholson et al., 2017). FIML has also been demonstrated as a viable method for handling missing values (Schlomer et al., 2010). Second, preliminary analysis, including descriptive statistics, bivariate correlations, and internal consistency reliability were conducted to provide basic demographic and variable-specific information (see Table 1). Third, confirmatory factor analysis (CFA) was conducted on the four scales mentioned above (i.e., school PsyCap scale, enjoyment subscale, anxiety subscale, and behavioral engagement scale) to establish construct validity. Fourth, a latent structural equation model (SEM) was applied to examine the relationships among these variables. The hypothesized model posited that school PsyCap (exogenous latent variable) would influence behavioral engagement (endogenous latent variable) through the mediators of academic enjoyment and anxiety (latent variables). Gender and prior mathematics achievement (observed variables) were included as covariates, given their documented associations with achievement emotions (e.g., Pekrun et al., 2017) and behavioral engagement (e.g., Wang et al., 2019) and that gender also result in differences in achievement emotions (Erturan and Jansen, 2015) and behavioral engagement (Nosek and Smyth, 2011). Finally, a bootstrapping procedure with 2,000 samples was employed to test the mediation effects (MacKinnon et al., 2004).

**TABLE 1:** Descriptive statistics, bivariate correlations, and internal reliability for key variables.

Variables	1	2	3	4	5	6
1. School PsyCap	–					
2. Math class-related enjoyment	0.540^**^	–				
3. Math class-related anxiety	−0.127^**^	−0.254^**^	–			
4. Behavioral engagement	0.592^**^	0.612^**^	−0.045	–		
5. Gender	−0.085^**^	−0.108^**^	0.225^**^	0.046	–	
6. Mathematics achievement	0.239^**^	0.370^**^	−0.153^**^	0.290^**^	0.028	–
Mean	3.23	3.041	3.206	3.403	1.490	0.000
SD	0.595	0.798	0.766	0.736	0.500	0.999
Cronbach’s α	0.97	0.83	0.66	0.78	–	–

Mathematics achievement was standardized z-scores.

^**^p < 0.01.

## 3 Results

### 3.1 Common method bias

This empirical study was based on participants’ self-reported data. Therefore, it is necessary to address common method bias before conducting the data analysis. To eliminate the potential common method bias, Harman’s single-factor test was employed (Podsakoff et al., 2003). The single-factor confirmatory factor analysis yielded a poor model fit: χ^2^ (324) = 2695.213, *p* < 0.001, RMSEA = 0.083, CFI = 0.750, TLI = 0.730, SRMR = 0.070. The findings demonstrate that common method bias was not expected to substantially impact the data analysis results.

### 3.2 Preliminary analysis

Table 1 presents the descriptive statistics and correlation coefficients among the study variables. In the analysis, school PsyCap was designated as the independent variable, while mathematics enjoyment and anxiety were viewed as mediators and behavioral engagement in mathematics classes as the dependent variable. Besides, gender and prior mathematics achievement were included as covariates.

### 3.3 Confirmatory factor analysis

Table 2 presents the goodness of fit indexes from CFAs of the study variables. Traditionally, the excellent and adequate cutoff values for model fit indexes were: (1) comparative fit index (CFI) and Tucker-Lewis index (TLI) are greater than or equal to 0.95 and 0.90, respectively; (2) the root mean square error of approximation (RMSEA) are less than or equal to 0.06 and 0.08, respectively; and (3) the values of standardized root mean square residual (SRMR) are less than or equal to 0.08 and 0.10, respectively (Chen, 2007; Hu and Bentler, 1999). Accordingly, the results indicated that a good model fit for all latent constructs was supported based on the conventional criterion of cutoff values.

**TABLE 2:** Confirmatory factor analysis (CFA) results of the four key variables.

Variables	χ^2^	df	CFI	TLI	RMSEA	SRMR
1. School PsyCap	406.672	86	0.936	0.922	0.061	0.036
2. Math class-related enjoyment	10.099	2	0.994	0.983	0.063	0.013
3. Math class-related anxiety	6.429	2	0.991	0.974	0.047	0.015
4. Behavioral engagement	13.097	2	0.990	0.969	0.074	0.017

### 3.4 Testing the hypothesized model

First, the associations among school PsyCap, behavioral engagement, and two covariates (i.e., gender and prior mathematics achievement) were tested. Results indicated good model fits, with χ^2^ (182) = 656.533, CFI = 0.930, TLI = 0.920, RMSEA = 0.051 and SRMR = 0.047. Significant associations between school PsyCap (β = 0.713, SE = 0.023 and *p* < 0.001) and behavioral engagement as well as gender (β = 0.122, SE = 0.028 and *p* < 0.001) and behavioral engagement were identified.

Second, the two mediators (i.e., academic enjoyment and anxiety) were added into the model. Results showed that the mediation model also has good model fits, with χ^2^ (362) = 1197.468, CFI = 0.914, TLI = 0.904, RMSEA = 0.048 and SRMR = 0.057 (Figure 1). Path one (school PsyCap → academic enjoyment → behavioral engagement) was significant (β = 0.339, SE = 0.030 and *p* < 0.001) for the 95% bootstrap bias-corrected confidence interval (BCa 95% CIs [0.285, 0.403]) did not contain zero. Path two (school PsyCap → academic anxiety → behavioral engagement) was also significant (β = −0.034, SE = 0.013 and *p* < 0.05) as (BCa 95% CIs [−0.065, −0.013]) did not contain zero. The significance of Path one and Path two indicated that academic enjoyment and anxiety mediate the relationship between school PsyCap and behavioral engagement. The association between school PsyCap and behavioral engagement, after taking mediators into consideration, decreased from β = 0.713 and *p* < 0.001 to β = 0.402 and *p* < 0.001, which revealed that academic enjoyment and anxiety act as partial mediators. Altogether, the indirect effect accounted for 48.1% of the link between school PsyCap and behavioral engagement, with a stronger link via academic enjoyment (43.7%) than via academic anxiety (4.4%), which could also be drawn from the small mediation of academic anxiety (β = 0.034).

Regarding the observed variables, results indicated prior mathematics achievement related positively to academic enjoyment (β = 0.163 and *p* < 0.001) and their behavioral engagement (β = −0.055 and *p* < 0.05) and negatively to academic anxiety (β = −0.138 and *p* < 0.001). Furthermore, the significant negative correlation between gender (male = 1 and female = 2) and academic enjoyment (β = −0.062 and *p* < 0.05) suggested that female students were less likely to take delight in mathematics lessons. While the significant positive correlation between gender and academic anxiety (β = 0.282 and *p* < 0.001) and behavioral engagement (β = 0.097 and *p* < 0.01) meant that female students were more engaged and anxious in mathematics classes.

## 4 Discussion

The present study investigated the emotional mechanisms linking school PsyCap to behavioral engagement in mathematics among secondary school students. A sample of Chinese seventh and eighth-grade students aged 12–15 years was surveyed to explore these associations. Controlling gender and prior mathematics achievement, SEM revealed that both positive and negative achievement emotions served as mediators in the relationship between school PsyCap and behavioral engagement. Supporting Hypothesis 1, higher levels of school PsyCap were positively correlated with academic enjoyment and negatively correlated with academic anxiety. In line with Hypothesis 2, the direct positive correlation between school PsyCap and behavioral engagement was also proved. Furthermore, findings aligned with Hypothesis 3, demonstrating that academic enjoyment and anxiety mediated the association between school PsyCap and behavioral engagement, accounting for a mediating effect of 48.1%. These findings highlight the importance of emotional factors in psychological pathways through which school resources influence student engagement in mathematics learning context.

School PsyCap is conceptualized as a promoter of students’ control appraisals, which in turn benefit the generation of achievement emotions (Kang and Wu, 2021; King and Caleon, 2021; Luthans and Youssef-Morgan, 2017). According to the CVT, control and value appraisals are the two proximal determinants of achievement emotions, while other distal antecedents (e.g., school PsyCap) act first on control or value appraisals and then on achievement emotions (Pekrun et al., 2011; Pekrun and Stephens, 2010). Thus, it is reasonable to expect that school PsyCap would be positively correlated with positive achievement emotions, such as academic enjoyment, and negative correlated with negative achievement emotions, such as academic anxiety. However, existing literature on school PsyCap was mainly conducted in Western contexts (e.g., Carmona-Halty et al., 2019, 2021), and only its correlation with positive emotions rather than negative emotions were discussed (Carmona–Halty et al., 2019; King et al., 2020). The present study advances the field by providing a more comprehensive examination of the relationship between school PsyCap and achievement emotions, explicitly incorporating negative emotions such as academic anxiety alongside positive ones. Comparison of the mediating effects revealed that the indirect influence of academic enjoyment on the relationship between school PsyCap and behavioral engagement (43.7%) was substantially greater than that of academic anxiety (4.4%). One possible explanation is that, with in Confucian heritage culture (CHC), positive emotions such as academic enjoyment are prioritized and reinforced, whereas negative emotions like academic anxiety are comparatively less emphasized (Pu, 2009). In the CHC context, virtues such as harmony, benevolence, and sincerity are highly valued (Ho, 2017; Shusterman, 2009), which to some extent facilitate the expression of positive emotions, such as academic enjoyment, while suppressing the outward manifestation of negative emotions like academic anxiety.

Multiple studies have evidenced that school PsyCap bolsters academic engagement among secondary school and college students (Kang et al., 2021; King et al., 2020; King and Caleon, 2021). Unlike previous research (e.g., You, 2016), the present study controlled for potential confounding variables such as gender and prior academic achievement to more accurately examine the relationship between school PsyCap and behavioral engagement. By accounting for these covariates, this study aimed to clarify the association between school PsyCap and behavioral engagement. However, beyond gender and prior mathematics achievement, additional factors such as socioeconomic status, age, parental involvement, and classroom environment may also influence the model structure by introducing potential biases in behavioral engagement or achievement emotions (Brumariu et al., 2023; Chen et al., 2021; Fiskerstrand, 2022; Mohammad Hosseini et al., 2022). While the present study contributes to existing research, future studies are advised to endeavor to control a broader range of variables to more comprehensively delineate the relationships among school PsyCap, achievement emotions, and academic engagement.

The primary purpose of the present study was to inspect whether achievement emotions mediated the relationships between school PsyCap and behavioral engagement in the mathematics context. Building on King et al. (2020) work, this study found that school PsyCap can affect behavioral engagement via both positive achievement emotion (i.e., academic enjoyment) and negative achievement emotion (i.e., academic anxiety). First, few studies have explored the emotional connection between school PsyCap and behavioral engagement (King et al., 2020, is an exception). Second, students experience both positive and negative achievement emotions in the learning process (Pekrun, 2006; Pekrun et al., 2011; Vierhaus et al., 2016; Westphal et al., 2018). However, in King et al.’s (2020) study, only the mediating effect of positive emotions have been confirmed. One possible reason is that their study was based on a sample of only 71 participants (King et al., 2020), and that small sample sizes might lead to mediating effect bias (MacKinnon et al., 2004). Third, King et al. (2020) did not control for prior academic achievement and the domain specificity of achievement emotions and behavioral engagement were also ignored. Thus, this is no way to determine whether school PsyCap offers incremental benefits over prior achievement in behavioral engagement. By controlling for gender and prior mathematics achievement and limiting the present study to the mathematics classroom context, it can be confidently concluded that the predictive power of school PsyCap on behavioral engagement is not an artifact of prior achievement. Furthermore, this study extended the investigation that school PsyCap, being a force “drives students to higher levels of flourishing” (King and Caleon, 2021, p.362), promotes behavioral engagement via a broader range of emotional mediators than in previous studies.

### 4.1 Theoretical implications

The present study simultaneously considered the antecedents and consequences of achievement emotions, thereby providing empirical support for the control-value theory and establishing an integrative framework for the comprehensive analysis of achievement emotions. The content of the CVT comprises two parts: on the one hand, control and value appraisals are the proximal antecedents of achievement emotions; other proximal antecedents of achievement emotions (e.g., school PsyCap) influence achievement emotions indirectly through control and value appraisals (Kang et al., 2025; Pekrun, 2006, 2019). On the other hand, achievement emotions have a significant impact on academic outcomes, including academic engagement, learning motivation, and academic performance (Earl et al., 2024; Kang and Wu, 2022a; Muwonge et al., 2018). In contrast to prior research that has primarily focused on either the antecedents or the consequences of achievement emotions (Kang and Wu, 2022b; Wu and Yu, 2022), the present study adopted an integrative approach by examining both aspects concurrently. This study proposed the “school PsyCap → achievement emotions → academic engagement” model, thereby facilitating a more comprehensive perspective for understanding achievement emotions.

### 4.2 Educational implications

This study contributes to the body of literature (Carmona–Halty et al., 2019; Datu et al., 2018; Datu and Valdez, 2016; Kang et al., 2021; King et al., 2020; Siu et al., 2014), suggesting that school PsyCap is adaptive for students’ behavioral engagement via the emotional mediators of academic enjoyment and anxiety. Teachers can implement a variety of measures to improve the level of their students’ school PsyCap. Given that the four higher-order core factors of school PsyCap (i.e., hope, self-efficacy, resilience, and optimism) are open to development, intervention training programs such as expanding students’ personal resources to facilitate them to overcome learning obstacles, setting up stepwise techniques and timelines to help students achieve their learning goals, identifying learning obstacles in advance and proposing corresponding solutions to increase students’ expectations for academic success are the effective ways for teachers to develop their students’ school PsyCap (see Luthans et al., 2010). Second, teachers are recommended to create a cooperative classroom atmosphere and to give full play to the roles of study groups and peer leaders as students’ self-confidence can be effectively boosted (Luthans et al., 2014), and thus the level of their school PsyCap was raised. Third, group-based acceptance and commitment therapy, which emphasizes mindfulness, acceptance, values, and cognitive defusion, has also been shown to boost adolescent students’ PsyCap (Fang and Ding, 2020). This indicates that teachers can help by creating a therapeutic classroom climate in which students’ PsyCap level is improved. Fourth, compared to their male counterparts, female students exhibited greater behavioral engagement in the mathematics classrooms. However, female mathematics learners experienced lower levels of enjoyment and higher levels of anxiety, suggesting that mathematics educators should provide greater support and care for female students to help mitigate their negative emotional experiences. Fifth, the mediating influence of academic enjoyment in the relationship between school PsyCap and behavioral engagement was remarkably pronounced than that of academic anxiety. Mathematics educators are suggested to prioritize strategies aimed at fostering students’ positive emotional experiences in learning mathematics, rather than exclusively focusing on the reduction or remediation of negative emotions. This proposition is consistent with the principles of the positive psychology movement within educational contexts. Mathematics educators could significantly enhance students’ positive emotional experiences by providing increased attention and care, fostering harmonious teacher-student relationships, and offering timely academic and emotional support. Such interventions are likely to exert a substantial positive influence on academic outcomes, such as academic engagement.

### 4.3 Limitations and directions for future research

This empirical study investigated the emotional connections of school PsyCap with behavioral engagement in mathematics classrooms of Chinese secondary school students, covering both positive and negative achievement emotions. Nevertheless, four potential limitations need to be acknowledged. First, only gender and prior academic achievement were controlled for in the present study, and it is suggested that more possible covariates should be considered in future studies. Family obligations (King and Ganotice, 2015), interpersonal relationships (Collie et al., 2016), family socioeconomic status (Chen et al., 2021) and classroom social climate (Mohammad Hosseini et al., 2022), for example, have also been shown to have an impact on the levels of behavioral engagement, indicating that future studies could deepen the understanding of the linkage between school PsyCap and behavioral engagement by way of controlling more antecedents of behavioral engagement. Second, a rigorous causal relationship between school PsyCap and behavioral engagement could not be determined because of the cross-sectional design of the present study. A longitudinal design is suggested to establish causality between these two constructs. Third, the data in the present study were all self-reported by the participants. Although common method bias has been addressed, future research is recommended to collect data from a broader range of resources, such as participants’ parents, teachers, and peers, to overcome the potential response bias effect (Conway and Lance, 2010). Fourth, although the Cronbach’s alpha values for academic anxiety, optimism, and self-efficacy were acceptable, they were not considered good. Thus, future research is recommended to undertake item refinement to enhance the reliability of these scales.

## 5 Conclusion

The present study demonstrated in a sample of 1135 Chinese secondary school students aged 12–15 years that school PsyCap predicts behavioral engagement via both positive and negative achievement emotions (i.e., academic enjoyment and anxiety). After controlling gender and prior mathematics achievement, students with a high level of school PsyCap showed higher positive achievement emotion and lower negative emotion in mathematics classes and subsequently showed more positive classroom engagement than students with a low level of school PsyCap. The school PsyCap level of students can be improved by teachers promoting intervention training programs and acceptance and commitment therapy, and that the classroom climate is therapeutic and cooperative.

## Data Availability

The raw data supporting the conclusions of this article will be made available by the authors, without undue reservation.
